# Supplementary material for: Extraordinarily long duration of Eocene geomagnetic polarity reversals
Source: Commun Earth Environ. 2026 Jan 20;7(1):180. doi: 10.1038/s43247-026-03205-8 (PMC12913026; doi:10.1038/s43247-026-03205-8)
Supplement: Supplementary file 2 — Supplementary Information [file 43247_2026_3205_MOESM2_ESM.pdf]

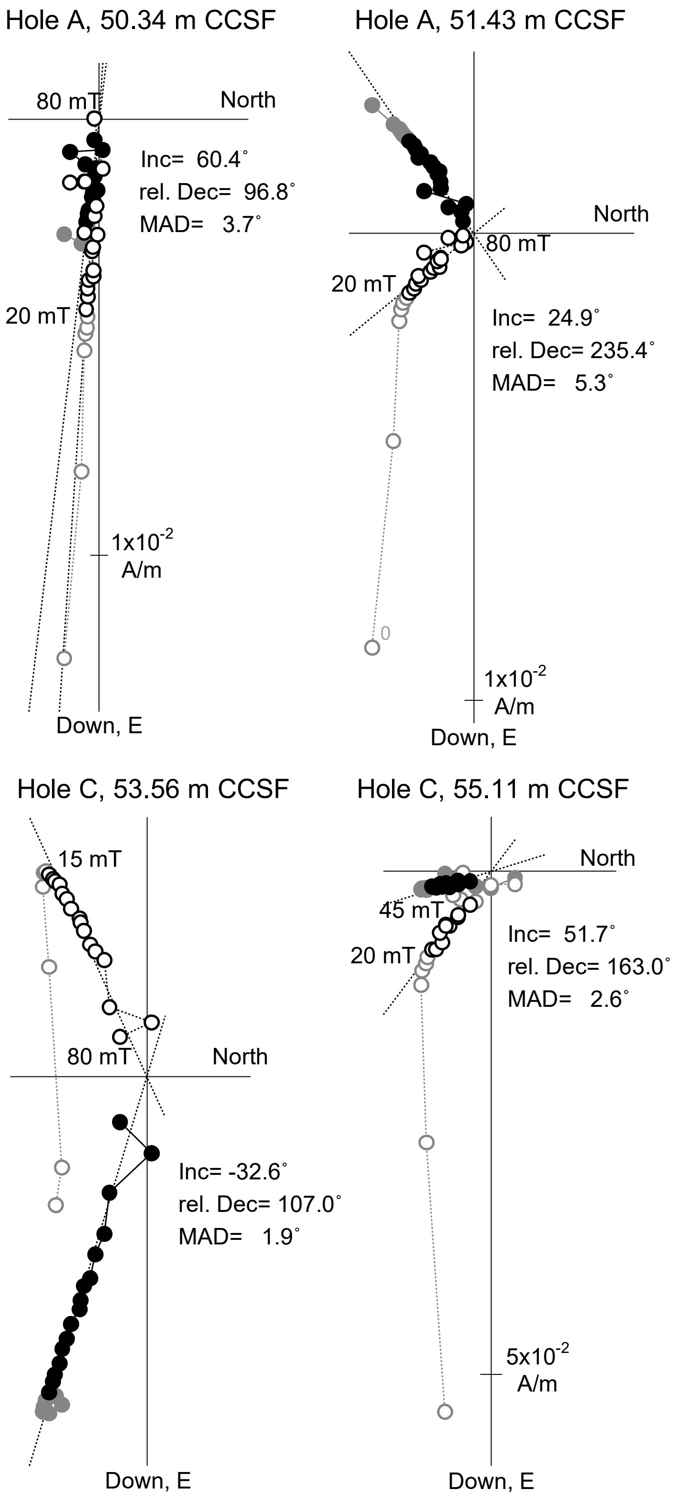


**Extended Data Fig. 1 | Representative orthogonal vector component plots of AF demagnetization results for the NRM of the studied samples.** Solid (open) circles indicate horizontal (vertical) projections. Grey circles represent data not used for PCA. Black dashed lines are best-fits to the data.

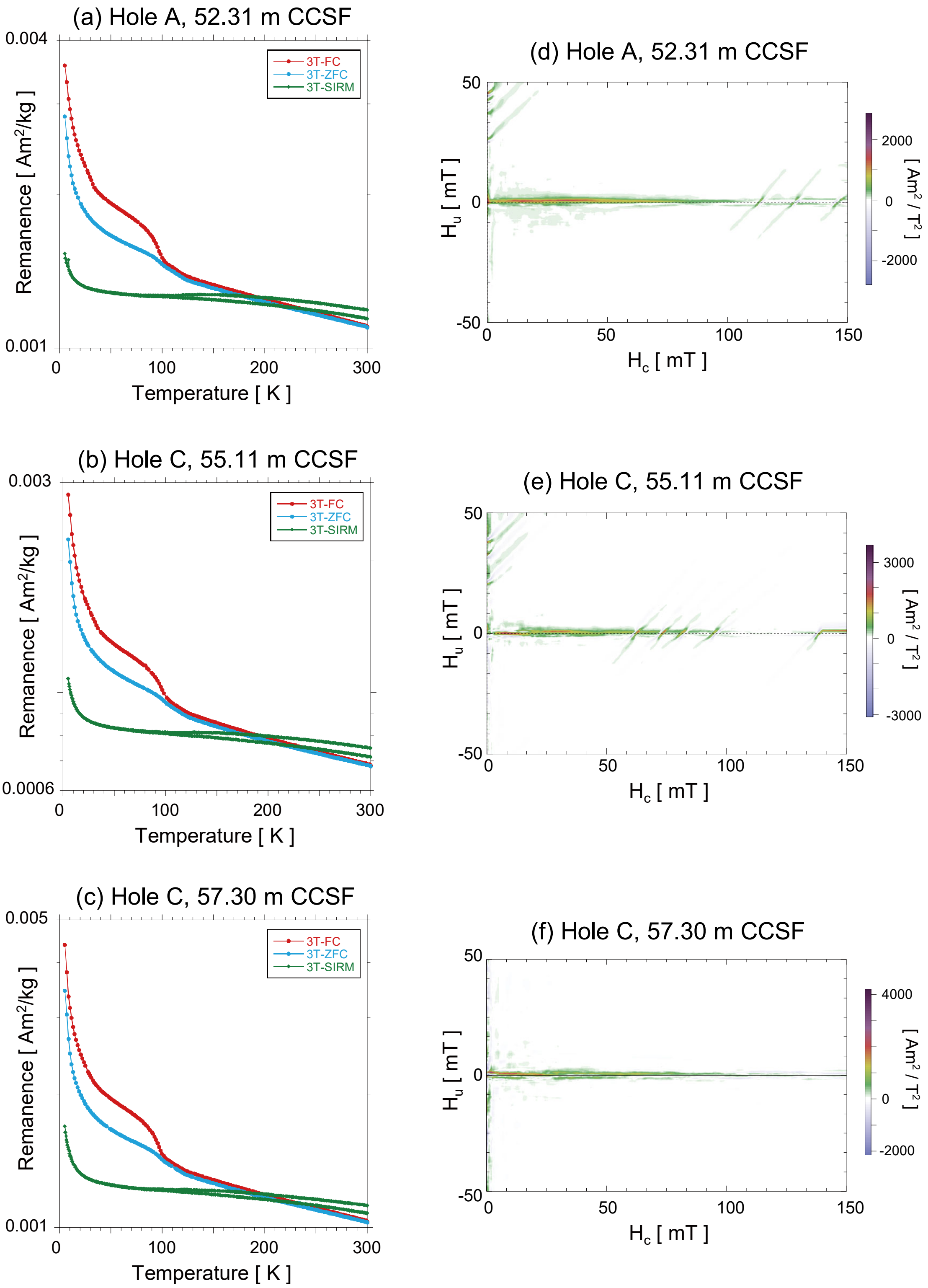


**Extended Data Fig. 2 | Representative results of low-temperature magnetometry.** They are shown for sample fractions selected from three horizons (a-c) and FORC diagrams for the same horizons (d-f).

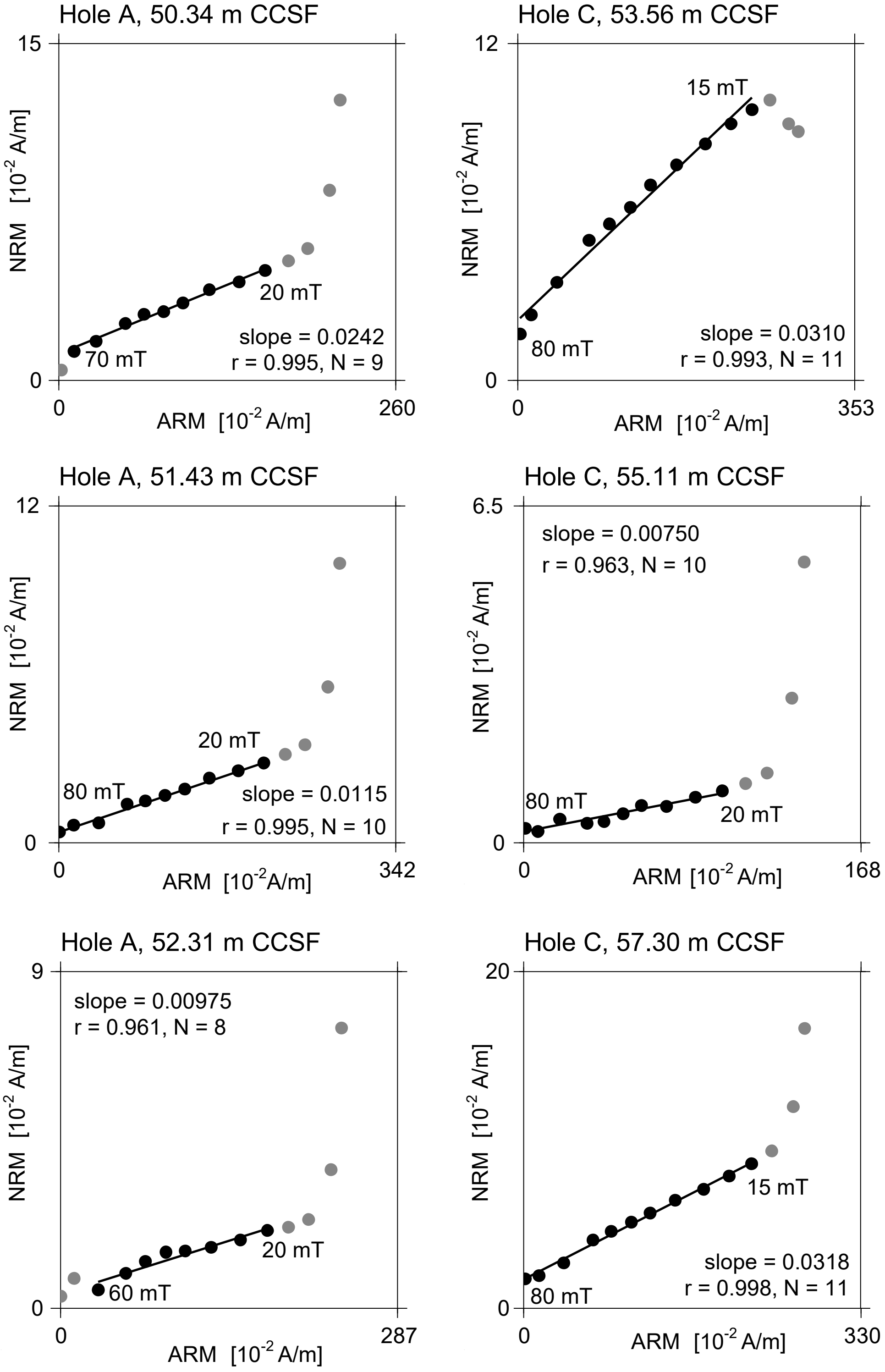


**Extended Data Fig. 3 | Representative NRM-ARM diagrams for AF demagnetization results for the studied samples.** Best-fit slopes are determined and their values with correlation coefficient (r) and number of data points (N) are indicated

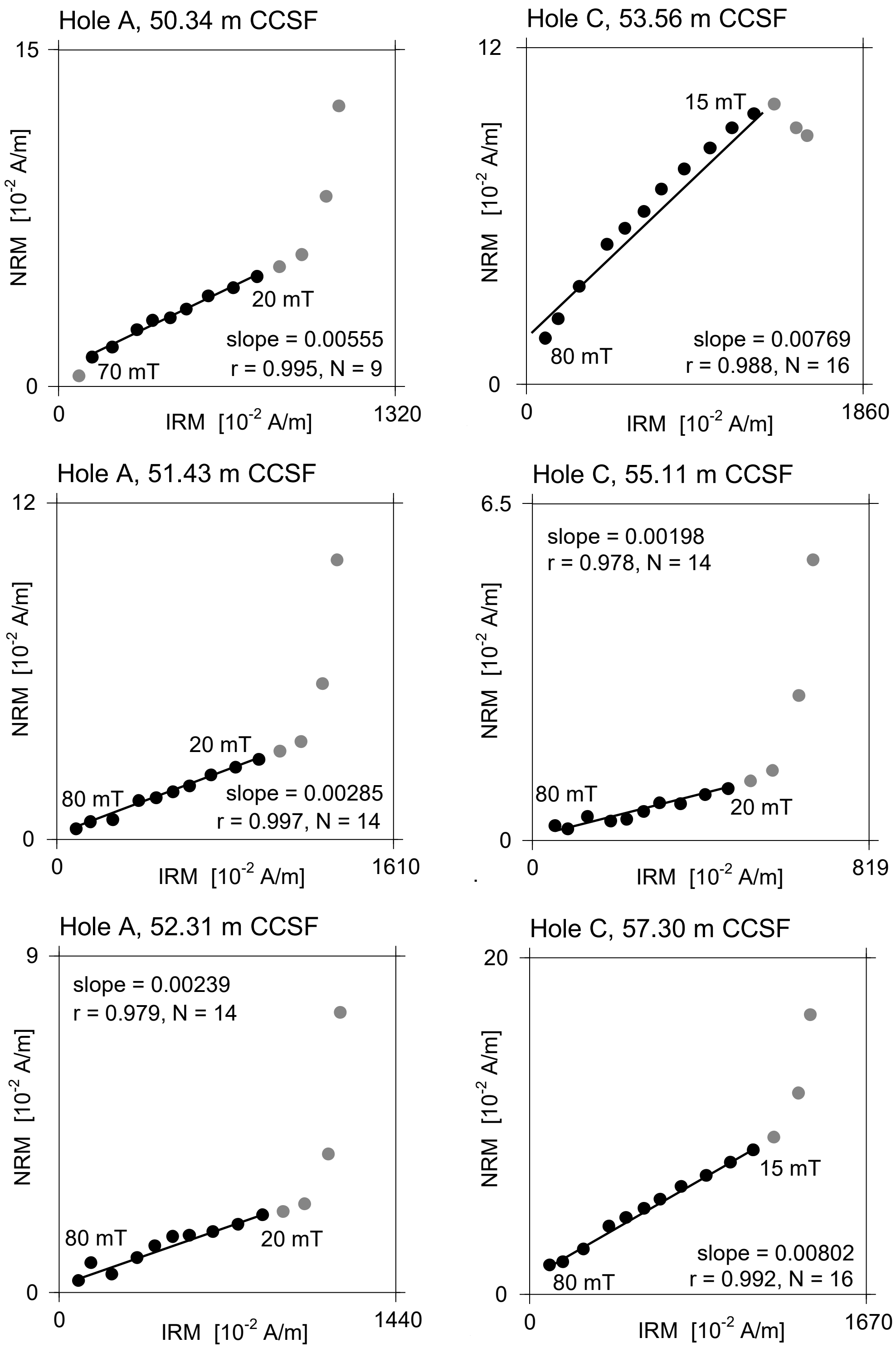


**Extended Data Fig. 4 | Representative NRM-IRM diagrams for AF demagnetization results from the studied samples.** Best-fit slopes are determined and their values with correlation coefficient (r) and number of data points (N) are indicated.

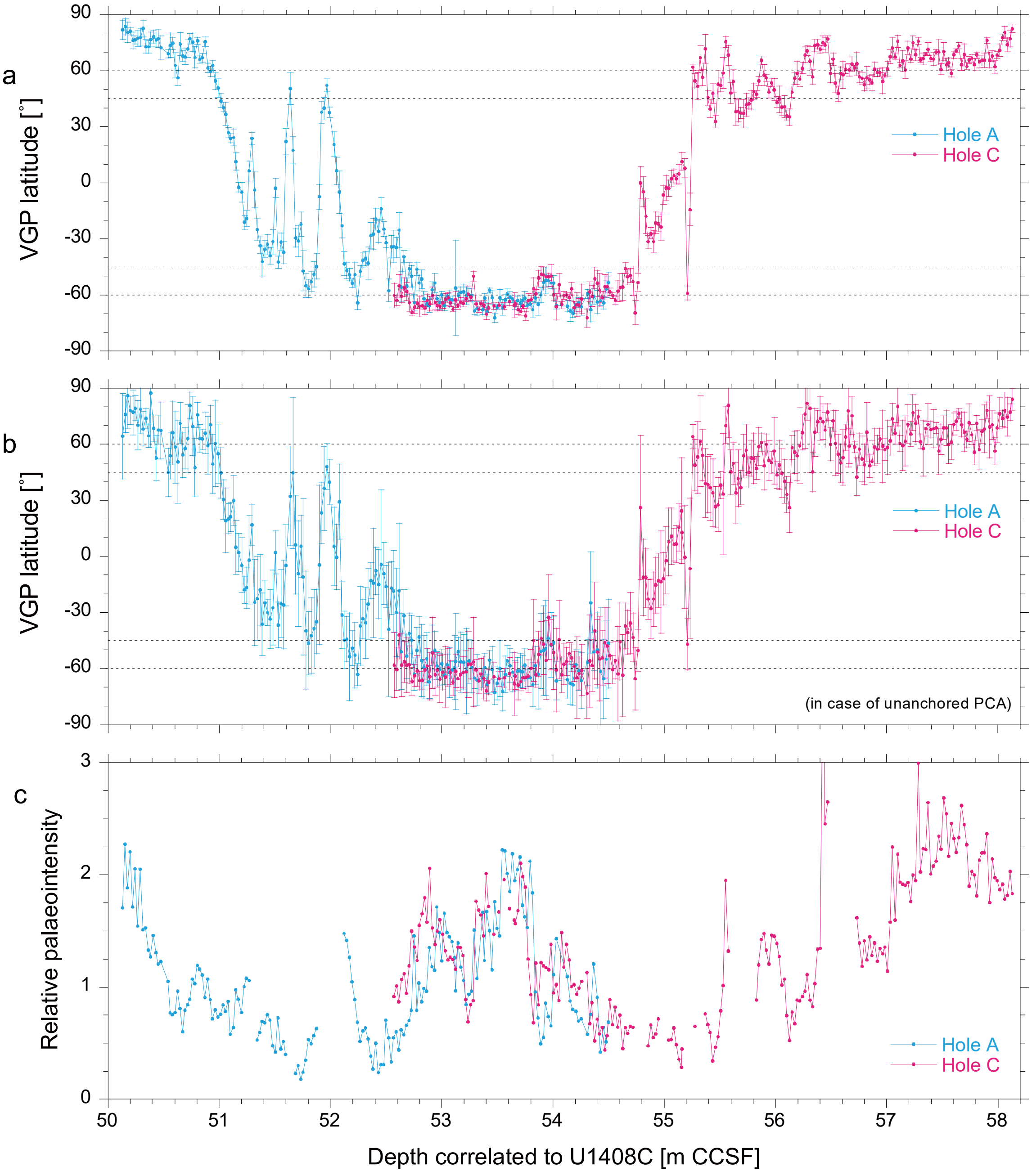


**Extended Data Fig. 5 | Downcore variations of VGPs and RPI plotted on the common depth scale.** VGP variations are shown with uncertainty proxies (MAD) based on PCA, with and without anchoring the fit to the origin, in panels 'a' and 'b', respectively. RPI variation is indicated in panel 'c'. The between-hole composite depth scale in meters CCSF was further adjusted to have the best matches in variations between Hole A (blue) and Hole C (red). Hole A depths were correlated to Hole C depths ($CCFS_{\text{Hole C}}$) by shifting the Hole A record 4 cm upward ($CCFS_{\text{Hole A}}$ - 0.04 m).

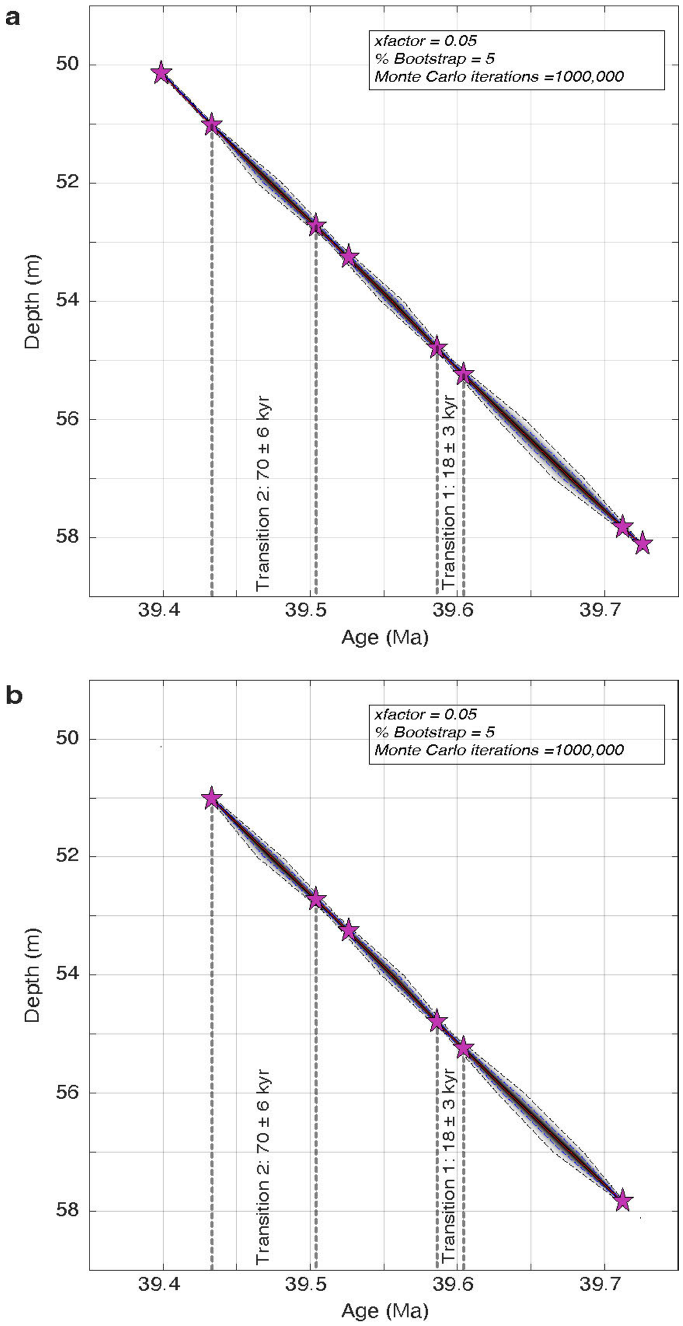


**Extended Data Fig. 6 | Age-depth model of the studied Eocene stratigraphic interval using the *"Undatable"* age-depth modeling routine[32].** The eight selected age-depth points (indicated by purple stars) are from younger to older ages: top of the studied stratigraphic interval, base of C18n.1n, base of transition 2, base-minimum of La2011 s3-s6 astronomical cyle No. 1229[18], base of C18n.1r, base of transition 1, base-minimum of La2011 s3-s6 astronomical cycle No. 1230[18], and base of the studied stratigraphic interval. (a) Using eight age-depth points. (b) Using six age-depth points (i.e., without the top and base of the stratigraphic interval). Uncertainties are provided with 1-sigma.

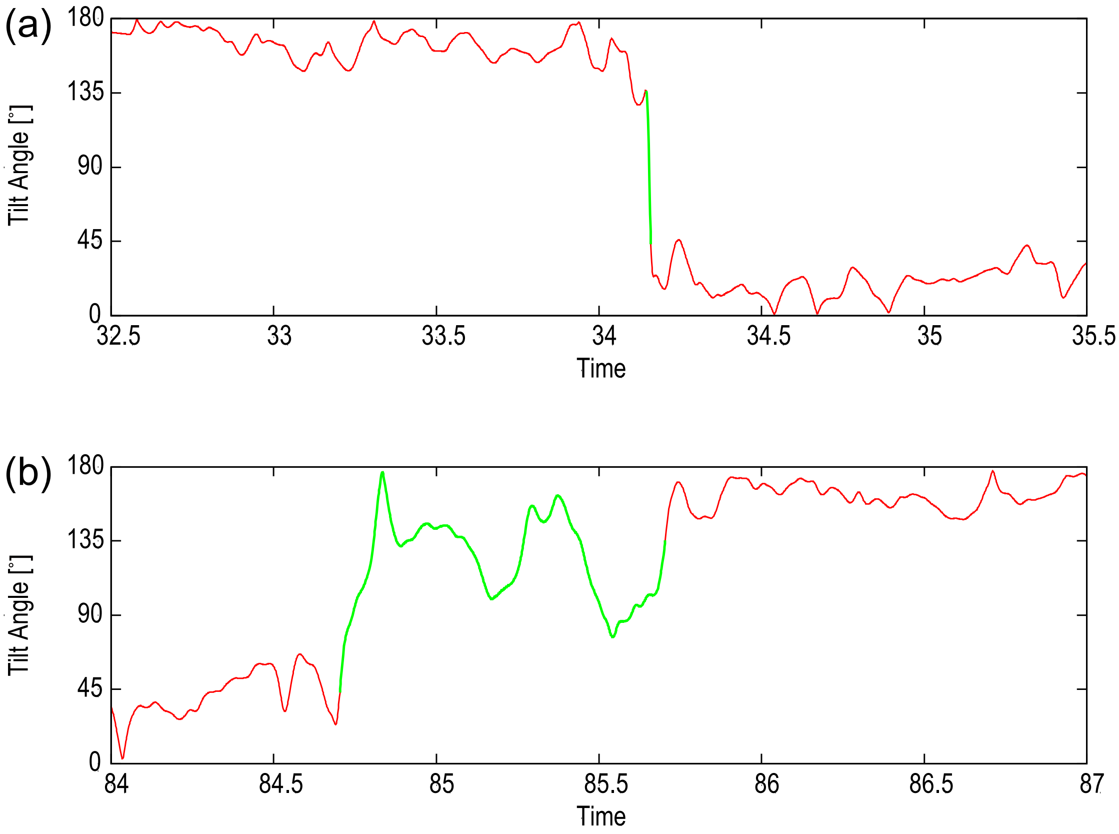


**Extended Data Fig. 7 | Typical examples of time-series for simple and complex polarity reversals in a numerical dynamo model (case-R2).** Simple and complex reversals are indicated in panels ‘a’ and ‘b’, respectively. Tilt angle represents dipole deviations from the rotation axis. Denoted in green is the duration of polarity transition according to the criteria adopted in this study.

**Extended Data Table 1 | Results of numerical geodynamo modeling.**

| Run ID | *Ek* | *Ra* | Total run time | Number of reversals | Mean duration | Range of durations | *Rm* | *Λ* |
|---|---|---|---|---|---|---|---|---|
| R1 | 0.00325 | 100 | 146.0 | 20 | 0.214 | [0.045, 1.016] | 167 | 8.8 |
| R2 | 0.00325 | 105 | 156.4 | 25 | 0.300 | [0.062, 1.441] | 172 | 9.5 |
| R3 | 0.00325 | 110 | 166.8 | 22 | 0.311 | [0.061, 1.598] | 178 | 9.4 |
| R4 | 0.00325 | 119.275 | 125.1 | 22 | 0.644 | [0.126, 3.298] | 194 | 8.1 |
| R5 | 0.002 | 180 | 127.3 | 15 | 0.194 | [0.044, 0.855] | 300 | 19.6 |
| R6 | 0.002 | 200 | 127.5 | 15 | 0.182 | [0.040, 0.822] | 326 | 17.2 |
| R7 | 0.002 | 220 | 135.5 | 20 | 0.275 | [0.054, 1.393] | 339 | 20.6 |
| R8 | 0.002 | 250 | 90.1 | 15 | 0.227 | [0.045, 1.156] | 372 | 17.8 |

Run ID: represents each dynamo case; *Ek*: Ekman number; *Ra*: Rayleigh number; Total run time: simulation run time in the unit of dipole free decay time; Mean duration: mean of the sampled reversal duration: Range of durations: minimum and maximum values of the reversal duration; *Rm*: Magnetic Reynolds number in terms of volume-averaged velocity; *Λ*: Elsasser number in terms of volume-averaged magnetic field.
